# Supplementary material for: Neolithic Yersinia pestis infections in humans and a dog
Source: Commun Biol. 2024 Aug 18;7:1013. doi: 10.1038/s42003-024-06676-7 (PMC11330967; doi:10.1038/s42003-024-06676-7)
Supplement: Supplementary file 2 — Supplementary Information [file 42003_2024_6676_MOESM2_ESM.pdf]

Supplemental Information for

Neolithic *Yersinia pestis* infections in humans and a dog

Julian Susat[1], Magdalena Haller-Caskie[1], Joanna H. Bonczarowska[1,2], Nicolas A. da Silva[1], Kerstin Schierhold[3], Michael M. Rind[3], Ulrich Schmölcke[4], Wiebke Kirleis[5], Holger Sondermann[6], Christoph Rinne[5], Johannes Müller[5], Almut Nebel[1], Ben Krause-Kyora[1]

[1]Institute of Clinical Molecular Biology, Kiel University, Kiel, Germany

[2]Department of Genetics, Yale School of Medicine, New Haven, CT, USA

[3]LWL-Archäologie für Westfalen, Münster, Germany

[4]Centre for Baltic and Scandinavian Archaeology (ZBSA), Schloss Gottorf, Schleswig, Germany

[5]Institute of Pre- and Protohistoric Archaeology, Kiel University, Kiel, Germany

[6]Centre for Structural Systems Biology (CSSB), Deutsches Elektronen-Synchrotron DESY, Hamburg, Germany

[*]Correspondence: b.krause-kyora@ikmb.uni-kiel.de

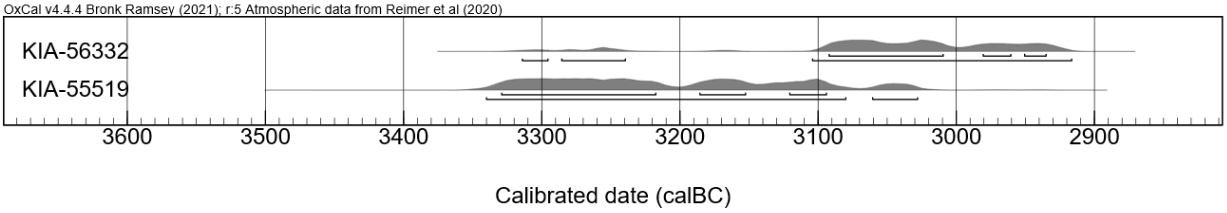

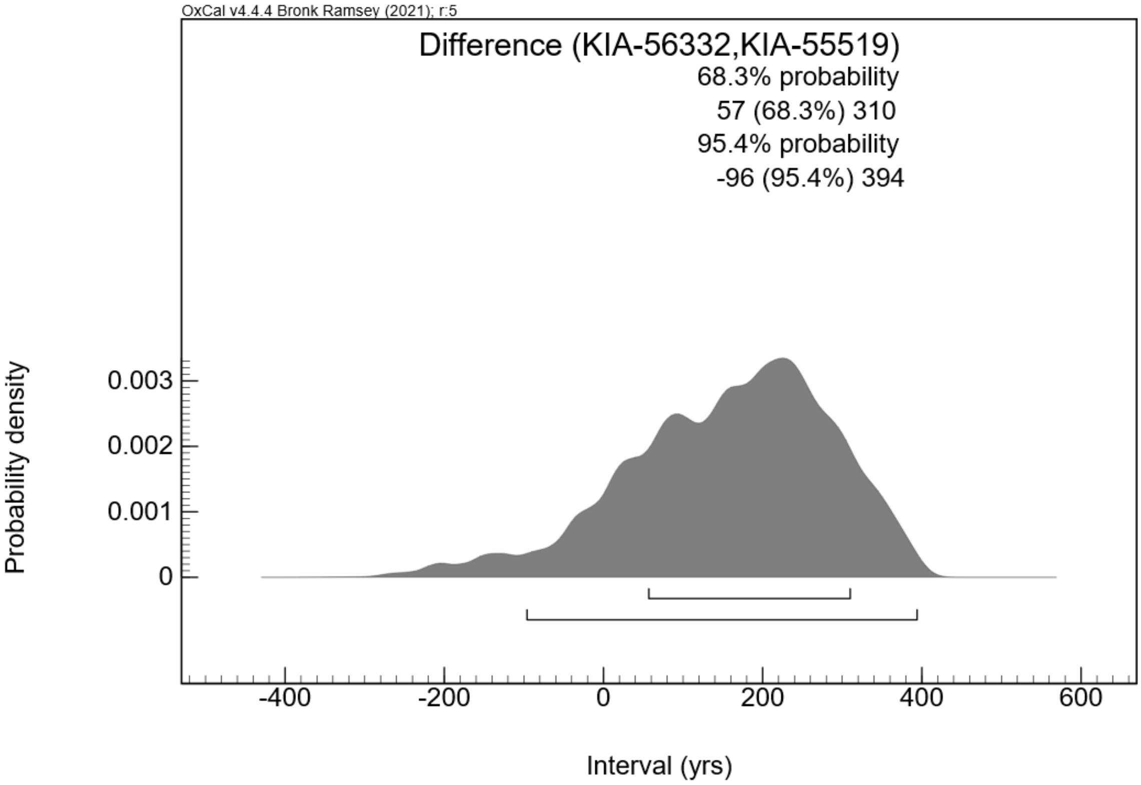

Supplementary Fig. 1. Radiocarbon dating results for Warburg_1 (KIA-55519) and Warburg_2 (KIA-56332), including the calculation of probability distributions for both samples.

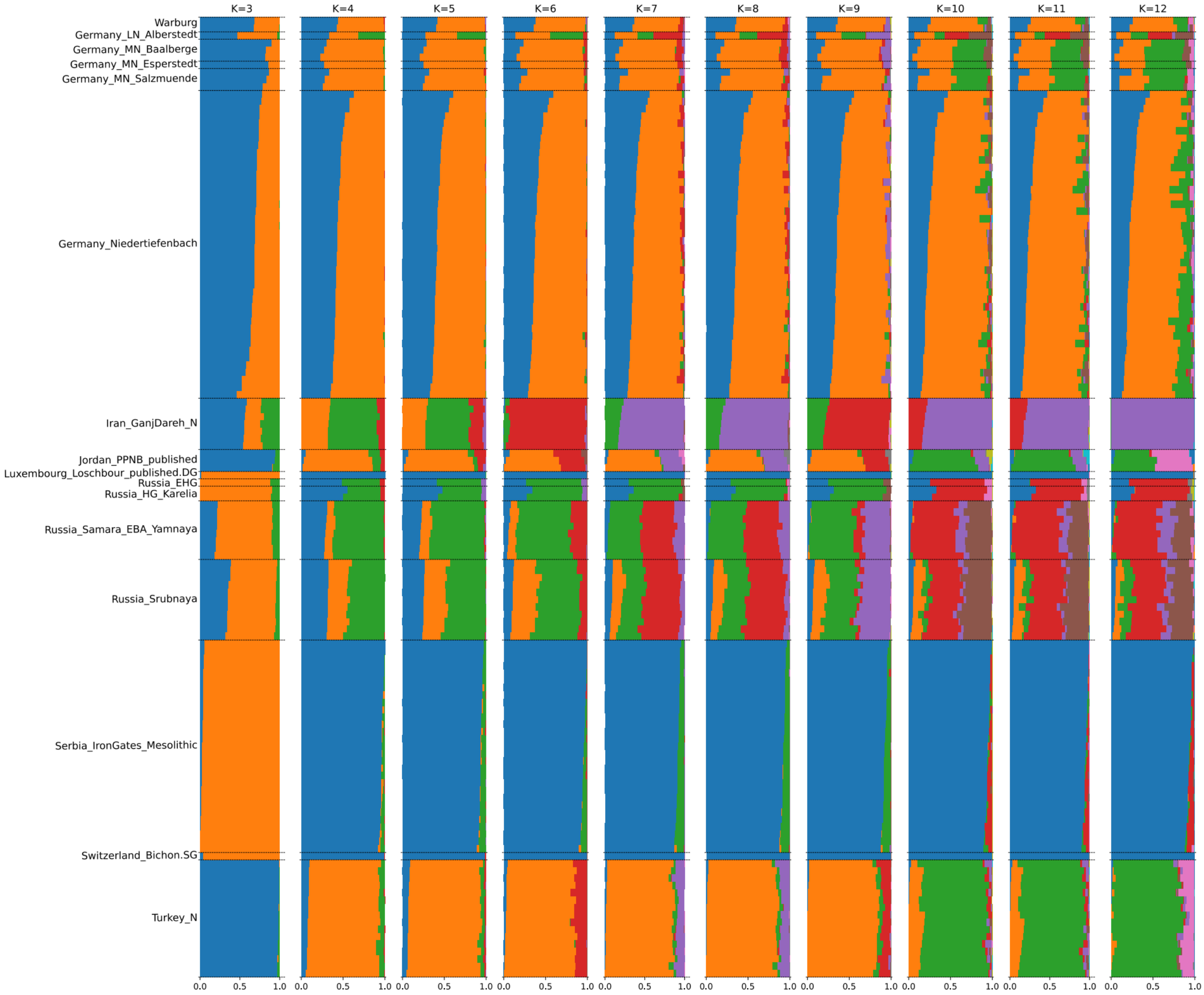

Supplementary Fig. 2. Genomic ancestry components using unsupervised ADMIXTURE. For the two Warburg individuals (red), most models indicate two major genetic components: one maximized in Western hunter-gatherers (i.e., Luxembourg_Loschbour) and the other in Anatolian farmers (i.e., Turkey_N).

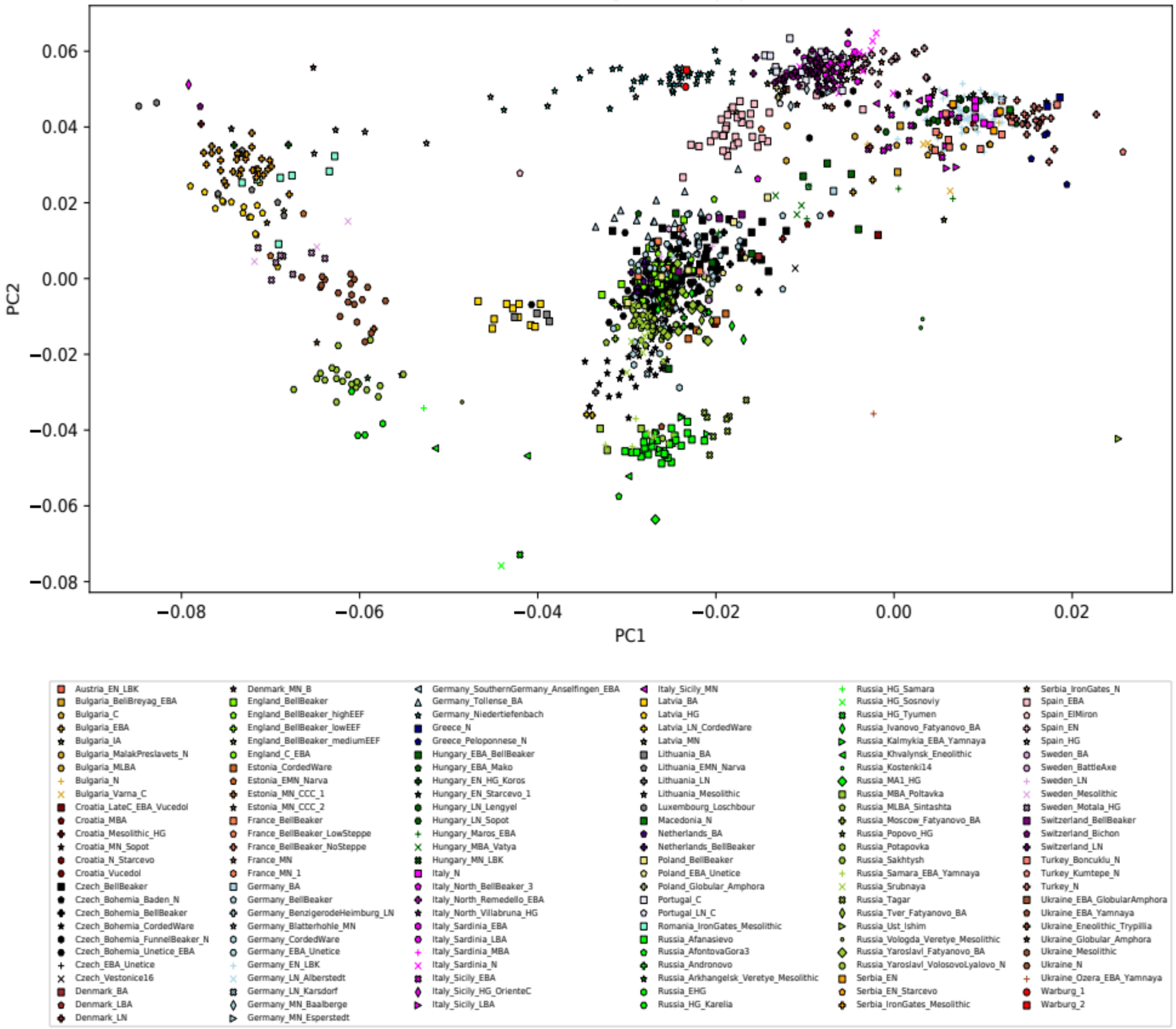

Supplementary Fig. 3. Principal component analysis of the individuals Warburg_1 and Warburg_2 buried in the WBC gallery graves of Warburg (red) in the context of 150 prehistoric populations, including individuals from the WBC gallery grave of Niedertiefenbach (blue stars).

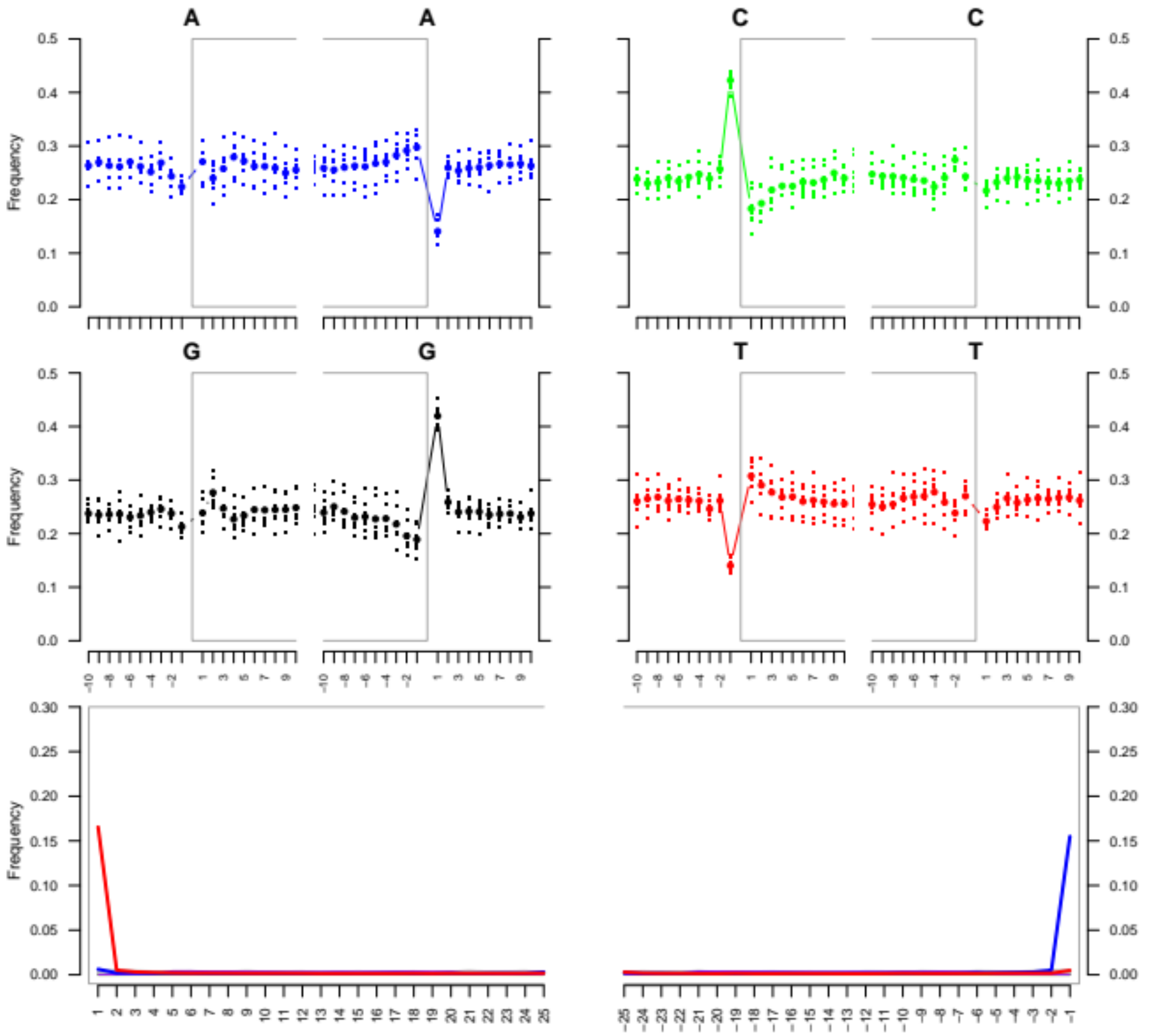

Supplementary Fig. 4. Damage plots supporting the ancient origin of *Y. pestis* reads for Warburg_1.

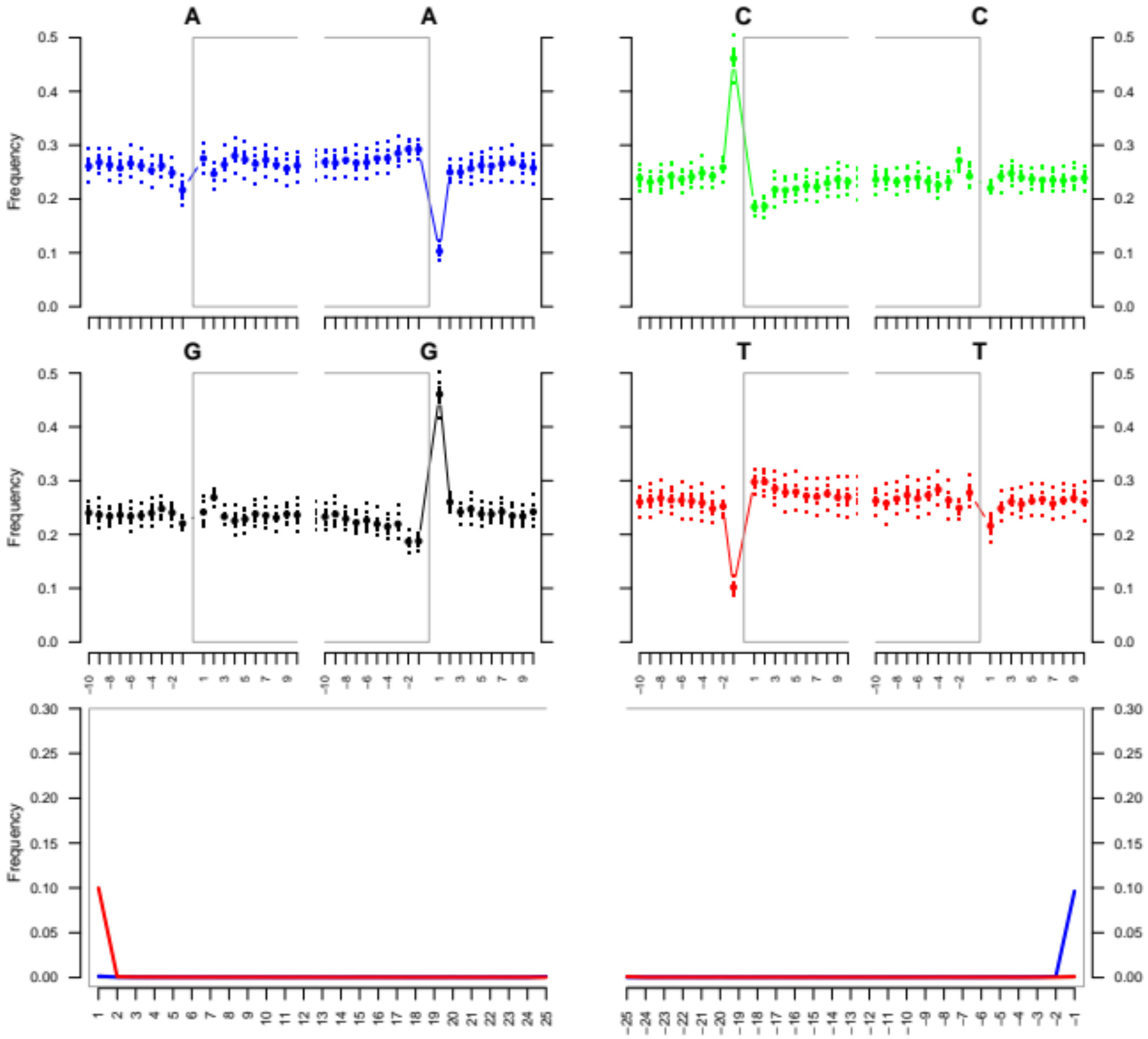

Supplementary Fig. 5. Damage plots supporting the ancient origin of *Y. pestis* reads for Warburg_2.

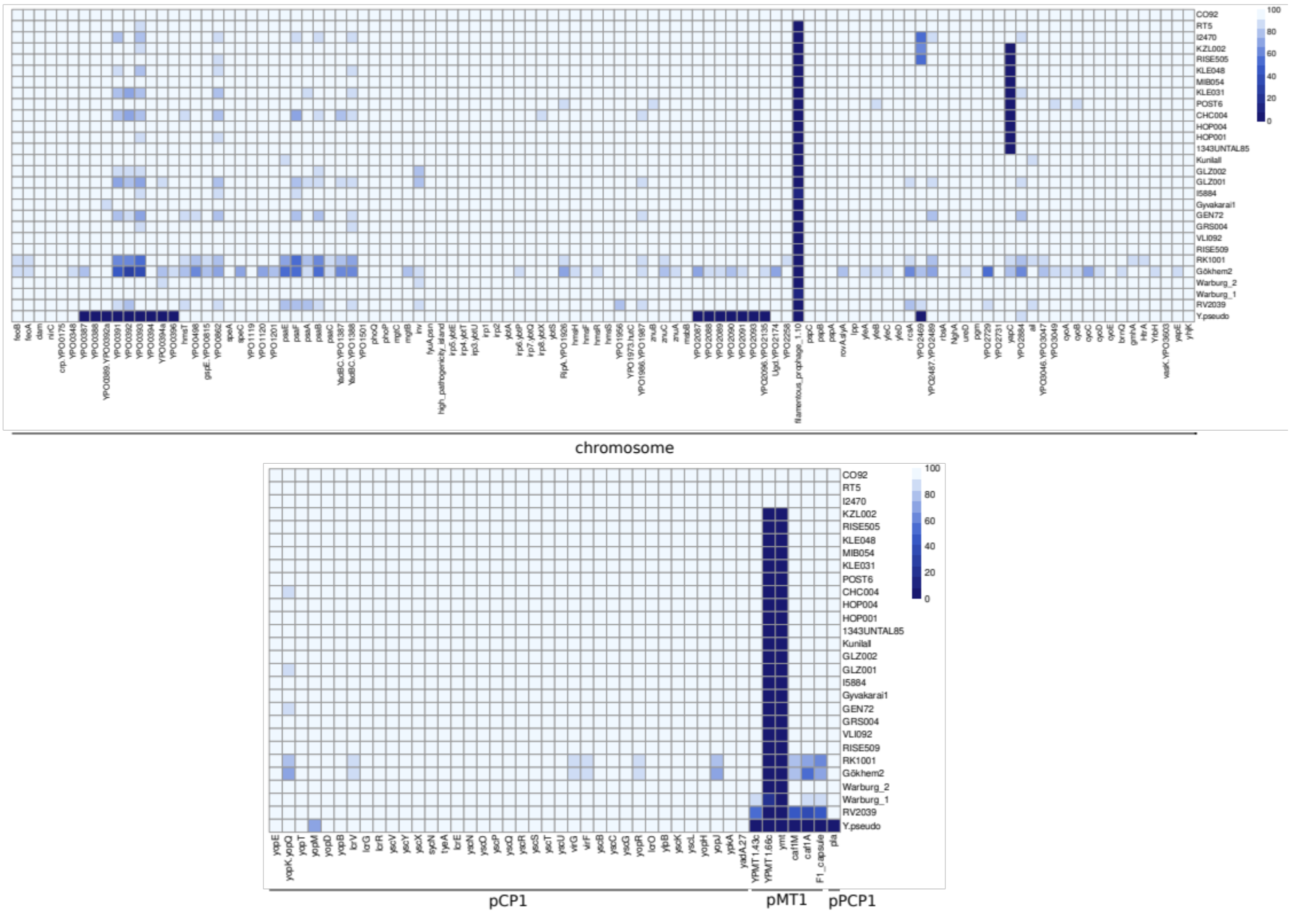

Supplementary Fig. 6. Visualization of virulence factors in ancient *Y. pestis* strains. Heatmap shows the presence or absence of 159 virulence factors on the chromosome (n=115) and the plasmids pCD1 (n=37), pMT1 (n=6) and pPCP1 (n=1). Genomes are sorted according to their placement in the phylogenetic tree (Fig. 2).

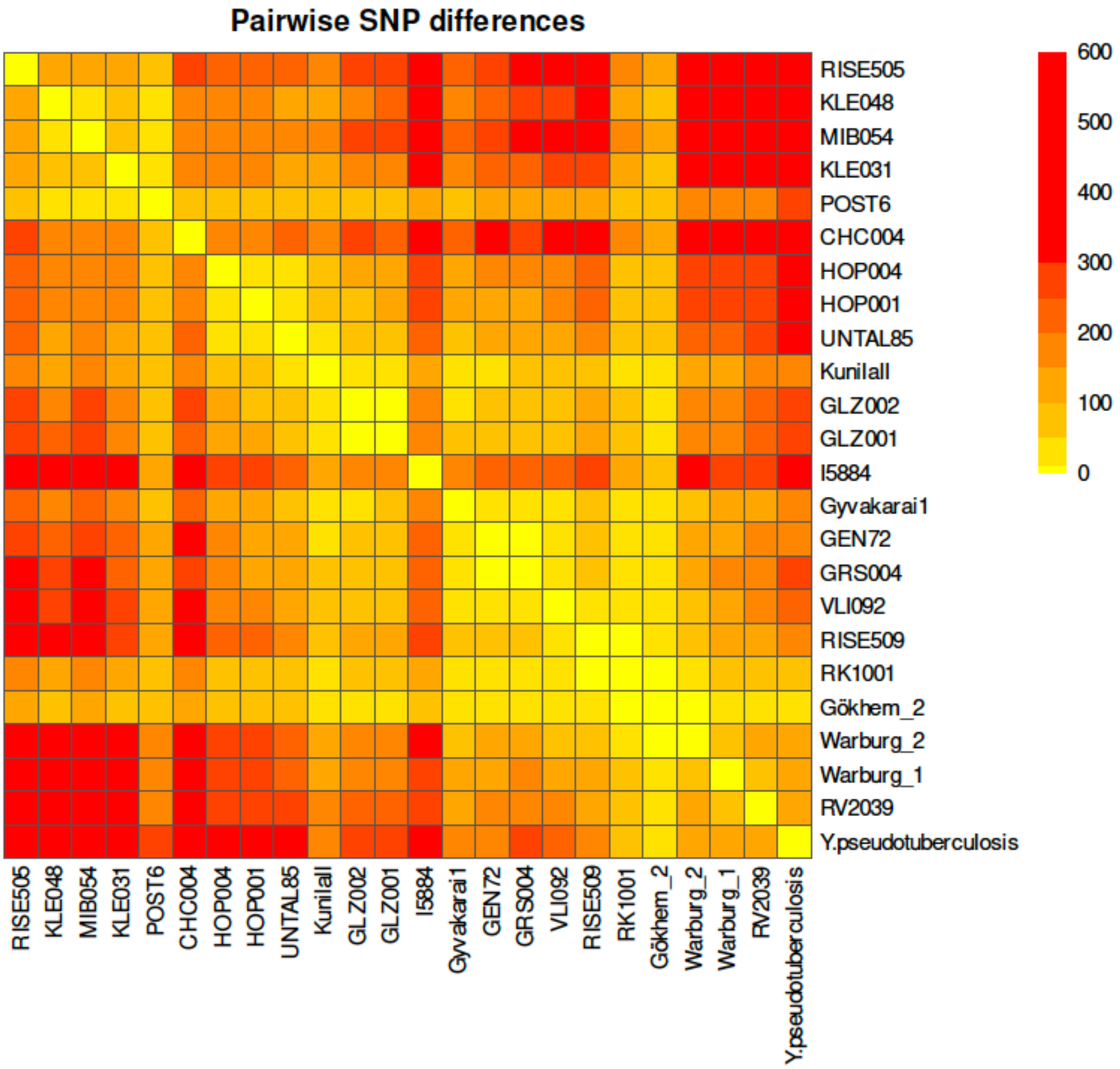

Supplementary Fig. 7. SNP distance matrix of LNBA and LN strains based on the Fasta alignments. Variants are counted if supported by a minimal coverage of 4 x.
