# Supplementary material for: Neolithic Yersinia pestis infections in humans and a dog
Source: Commun Biol. 2024 Aug 18;7:1013. doi: 10.1038/s42003-024-06676-7 (PMC11330967; doi:10.1038/s42003-024-06676-7)
Supplement: Supplementary file 3 — Description of Additional Supplementary Files [file 42003_2024_6676_MOESM3_ESM.pdf]

# Description of Additional Supplementary Files

File: Supplementary Data 1
Description: Yersinia pestis-positive Warburg individuals: General information on the sample material and mapping statistics (mapping to Yersinia pestis reference genome

File: Supplementary Data 2
Description: Yersinia pestis-positive Warburg individuals: Kinship inference with READ.

File: Supplementary Data 3
Description: Yersinia pestis-positive Warburg individuals: Analysis of genomic ancestry components using qpADM. Outgroups used: Mbuti.SDG, Russia_Ust_Ishim_HG_published.DG, Russia_Kostenki14, Russia_MA1_HG.SG, Han.SDG, Papuan.SDG, ONG.SG, Chukchi.DG, Karitiana.SDG.

File: Supplementary Data 4
Description: Table of nonsense and missense SNPs due to STOP/START codon gain/loss. Only

File: Supplementary Data 5
Description: Number of nucleotide positions differing between the Warburg genomes and those from the LNBA clade and the second pandemic.

File: Supplementary Data 6
Description: 226 modern and 64 ancient strains used in the SNP-based phylogeny, with Y.

File: Supplementary Data 7
Description: Subset of 42 strains used for molecular dating with BEAST2.
